# *Streptococcus pneumoniae *nasopharyngeal colonization induces type I interferons and interferon-induced gene expression

**DOI:** 10.1186/1471-2164-10-404

**Published:** 2009-08-27

**Authors:** Elizabeth A Joyce, Stephen J Popper, Stanley Falkow

**Affiliations:** 1 Department of Microbiology and Immunology, Stanford University School of Medicine, 299 Campus Drive, Stanford, CA 94131, USA

## Abstract

**Background:**

We employed DNA microarray technology to investigate the host response to *Streptococcus pneumoniae *in a mouse model of asymptomatic carriage. Over a period of six weeks, we profiled transcript abundance and complexity in the Nasal Associated Lymphoid Tissue (NALT) to identify genes whose expression differed between pneumococcal-colonized and uncolonized states.

**Results:**

Colonization with *S. pneumoniae *altered the expression of hundreds of genes over the course of the study, demonstrating that carriage is a dynamic process characterized by increased expression of a set of early inflammatory responses, including induction of a Type I Interferon response, and the production of several antimicrobial factors. Subsequent to this initial inflammatory response, we observed increases in transcripts associated with T cell development and activation, as well as wounding, basement membrane remodeling, and cell proliferation. Our analysis suggests that microbial colonization induced expression of genes encoding components critical for controlling JAK/STAT signaling, including *stat1, stat2*, *socs3*, and *mapk1*, as well as induction of several Type I Interferon-inducible genes and other antimicrobial factors at the earliest stages of colonization.

**Conclusion:**

Examining multiple time points over six weeks of colonization demonstrated that asymptomatic carriage stimulates a dynamic host response characterized by temporal waves with distinct biological programs. Our data suggest that the usual response to the presence of the pneumocccus is an initial controlled inflammatory response followed by activation of host physiological processes such as response to wounding, basement membrane remodeling, and increasing cellular numbers that ultimately allow the host to maintain an intact epithelium and eventually mount a preventive adaptive immune response.

## Background

*Streptococcus pneumoniae*, also known as the pneumococcus, is a Gram positive, encapsulated bacterium recognized as an important cause of pneumonia, meningitis and sepsis throughout the world. Despite its nefarious reputation, this organism usually establishes an obligate asymptomatic association within the human nasopharyngeal cavity. This carrier state is of critical importance since individuals asymptomatically colonized with *S. pneumoniae *serve as a reservoir for person to person transmission of this organism, highlighting the significance of the nasopharyngeal niche to pneumococcal survival within human populations. Asymptomatic pneumococcal colonization of the upper respiratory tract is prevalent in most human populations, with carriage rates exceeding 50% in children ≤ 1 year [1]. While more common in children, pneumococcal carriage in adults has been well documented [2]. Epidemiological evidence shows that the duration of carriage in most individuals is transient, typically lasting 4–12 weeks [3,4].

The mechanisms underlying the establishment and clearance of the pneumococcus from this niche are not well understood. Recent studies have shown that asymptomatic pneumococcal carriage induces localized mucosal and systemic cellular immune responses in both rodents and humans [5,6]. Subsequent clearance of the organism is dependent upon the presence of CD4+ T cells at the time of infection [7-9], suggesting a previously unrecognized and important role for the cellular arm of the immune response in the control of this pathogen.

The purpose of this study is to develop a picture of the host response that occurs during establishment and resolution of transient asymptomatic pneumococcal carriage in the nasopharyngeal cavity. Insight into the events that follow colonization may help us to better understand how the host response to this mucosal surface interaction develops. We performed a genome-wide transcriptional analysis of the Nasopharyngeal Associated Lymphoid Tissue (NALT) and overlying epithelial layer isolated from infected and mock-infected mice over a six-week period. Our analysis identified several hundred genes whose expression was significantly changed in *S. pneumoniae*-colonized mice. Most notably, the host response to pneumococcal carriage is characterized by innate immune responses involving early induction of Type I Interferons and Type I interferon-induced genes, hallmarks typically associated with intracellular bacterial and viral infections. These results suggest that these early innate responses may play an as yet unappreciated role in controlling asymptomatic mucosal colonization by *S. pneumoniae*.

## Results

### Pneumococcal colonization of the murine nasopharyngeal cavity

**Figure 1:**
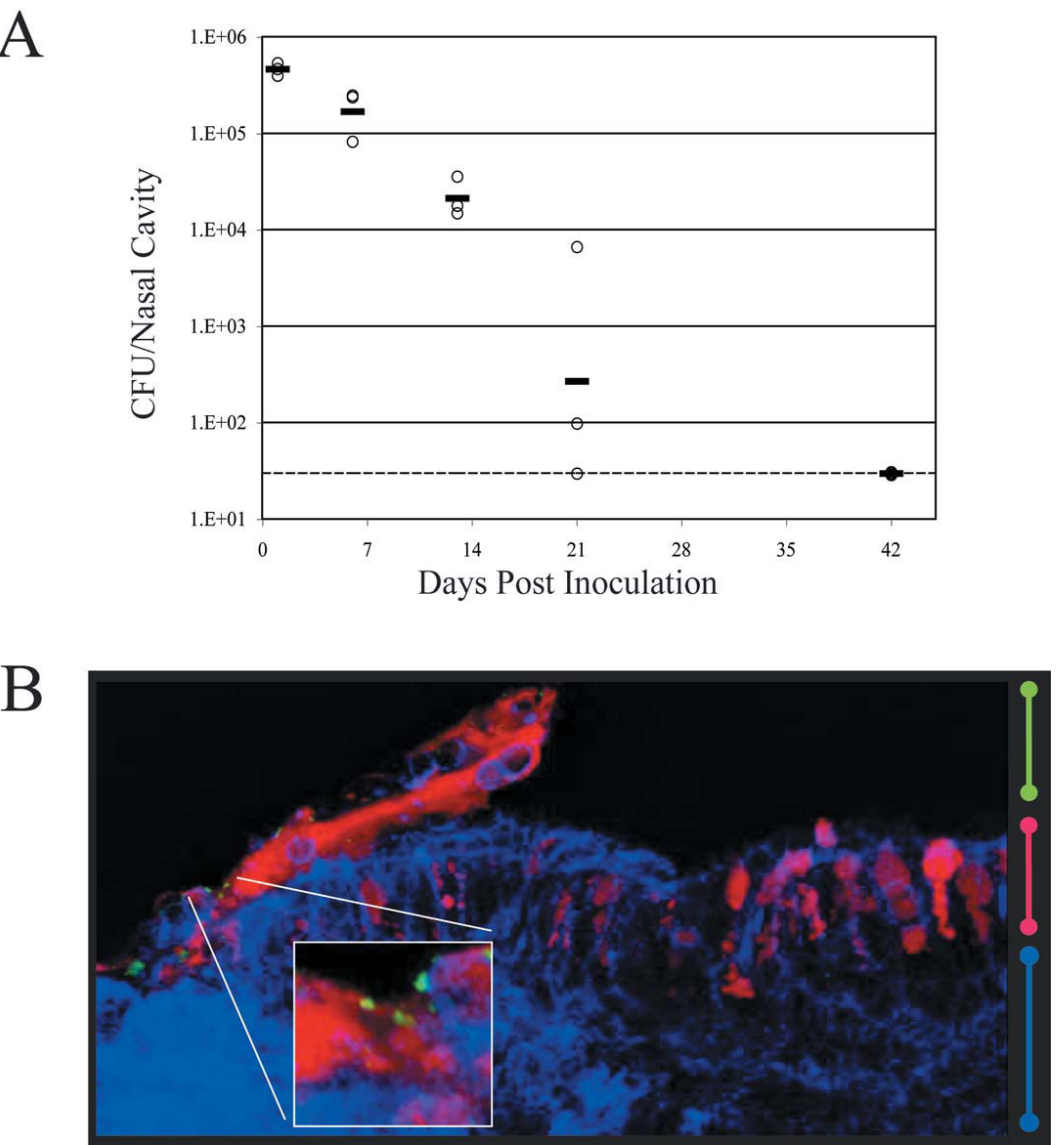
***S. pneumoniae *colonizes the nasopharynx of mice over a 6 week period**. **(A) **Viable cell counts from head cavities (post-NALT excision) of infected mice. Open circles represent viable counts from a single mouse. Solid lines represent the geometric mean of CFUs/head for each day. Dashed line represents the limit of detection. **(B) **Confocal immunofluorescence image of *S. pneumoniae *colonizing NALT tissue 3 days post-inoculation. The green bar bell indicates the lumen of the nasopharynx, the pink bar bell indicates the epithelial monolayer, and the blue bar bell indicates the cells within the NALT. *S. pnuemoniae *were visualized via fluorescently-conjugated antibody (green) near the surface of the NALT (blue) and were particularly apparent in the mucus layer (red).

Pneumococcal colonization of the murine nasal cavity has been previously demonstrated [10,11]. We colonized BALB/c mice intranasally with 2 × 10^8 ^Type 2 pneumococci and monitored carriage over a six-week period. At each time point, mice from both infected and mocked-infected groups were sacrificed and NALT tissue was excised as described [12]. NALT was chosen for host transcriptional analysis because it is the functional homolog of human tonsils and adenoids, which are thought to be important local inductive and effector sites for both mucosal and systemic responses to pneumococcal carriage [6,13,14]. The remainder of the head was homogenized in PBS, which was then serially diluted and plated to determine viable counts of the pneumococcus in the nasopharyngeal cavity. Figure 1A shows the pneumococcal load in the nasopharynx over a six-week period. Viable counts decreased approximately three logs over a three week period, and the bacteria were cleared by six-weeks post infection, as we were unable to detect viable pneumococci in the samples. In addition, fluorescence confocal microscopy of nasal tissue allowed us to visualize pneumococci at the NALT. Figure 1B shows a sagittal section through the NALT isolated from a mouse that had been inoculated with *S. pneumoniae *three days prior. Using a fluorescently conjugated antibody directed against the strain of *S. pneumoniae *used in these studies, we detected bacteria on the surface of the NALT.

### Gene expression patterns associated with pneumococcal colonization

We collected NALT samples from 3 infected and 3 mock-infected mice at each of 5 time points during the course of the experiment. Total RNA was isolated from these samples and was prepared for microarray analysis as described in the Material and Methods section. We used two different methods to identify genes whose patterns of expression distinguished infected and mock-infected samples. The first method, Significance Analysis of Microarrays (SAM) [15], identified genes whose expression consistently differed between infected and mock-infected mice over the entire time course. Of the 22,603 spots that met or exceeded our cutoff threshold for high quality data, this analysis identified 112 spots representing 87 genes (False Discovery Rate = 5%) whose level of expression was significantly associated with infection, and in 111/112 cases, transcript levels were more abundant in the colonized animals (See Additional file 1: Table S1). Since we hypothesized that carriage is a dynamic process, we were also interested in identifying those genes whose expression changes over the course of the infection. Thus, in a second type of data analysis, we assessed the same list of 22,603 spots by quantifying the total amount of variation in expression observed for each gene in both the infected and mock-infected groups [16]. 7,163 spots were determined to show greater variance in the infected mice as compared with the mock-infected controls. These spots were ranked from most variable to least variable and we considered the top 5% most variable data (358 spots) for further analysis (see Additional file 2: Table S2).

### Ontological analysis of the gene lists reveals several significant biological themes

The list of genes that distinguish infection from mock-infection defined by the SAM and variation analyses were further analyzed to identify biological themes associated with them. To classify genes into categories, we employed GeneTrail, a web-based software application that evaluates gene lists for statistically significant enrichment of biological processes when compared to the reference input list [17,18]. To identify lexical enrichments, this program uses terminology defined by the Gene Ontology (GO) Consortium [19,20] and the Kyoto Encyclopedia of Genes and Genomes (KEGG) database [21,22]. The genes listed in Table 1 were identified in the SAM analysis and are associated with three GO categories: Mitosis/Cell Division (*p *≤ 0.003), Microtubule-based Process (*p *≤ 0.035), and Immune System Process (*p *≤ 0.051). Table 2 lists the processes that were enriched in the Variance gene list, which includes the JAK/STAT Signaling Pathway (*p *≤ 0.09), Basement Membrane (*p *≤ 0.04), Response to Wounding (*p *≤ 0.03), DNA Replication (*p *≤ 0.03), and Cell Division/Cell Cycle (*p *≤ 0.02).

**Table 1:** Over-represented GO categories and the associated genes identified by GeneTrail from the SAM data.

**Mitosis/Cell Division** **(p ≤ 0.003)**	**Microtubule-based** **Process (p ≤ 0.035)**	**Immune System Process** **(p ≤ 0.051)**
*ccng1*	*cenpe*	*flt3*

*cdc25c*	*fbxo5*	*il1rl1*

*cdc45l*	*nuf2*	*lilrb4*

*cdca5*	*tuba3a*	*msh6*

*chek1*	*xrn2*	*pou2f2*

*fbxo5*		*ptpn22*

*nuf2*		*tacc3*

*sgol1*		*vav1*

*tipin*		*vav2*

**Table 2:** Over-represented categories and the associated genes identified by GeneTrail from the variance data.

**JAK/STAT** **(p ≤ 0.09)**	**Basement Membrane** **(p ≤ 0.04)**	**Response to Wounding** **(p ≤ 0.03)**	**DNA Dependent DNA Replication** **(p ≤ 0.03)**	**Cell Cycle/Cell Division** **(p ≤ 0.02)**
*ccnd1*	*adamts*	*cd55*	*gmnn*	*bub1*

*il10ra*	*col8a1*	*fn1*	*clspn*	*ccnd1*

*stat1*	*entpd1*	*myh10*	*prim2*	*cdk8*

*stat2*	*lad1*	*reg3g*	*mcm6*	*cdkn2a*

	*nid2*	*f3*	*mcm7*	*clspn*

		*entpd1*		*fert2*

		*ccl24*		*gas1*

		*gja1*		*gmmn*

		*chi3l3*		*hells*

		*darc*		*mad2l1*

				*mapk1*

				*mcm6*

				*mcm7*

				*mki67*

				*mphosph1*

				*mtbp*

				*nasp*

				*ncapg2*

				*sept6*

				*syce2*

				*tlk1*

### Dynamic changes in gene expression over the course of infection

**Figure 2:**
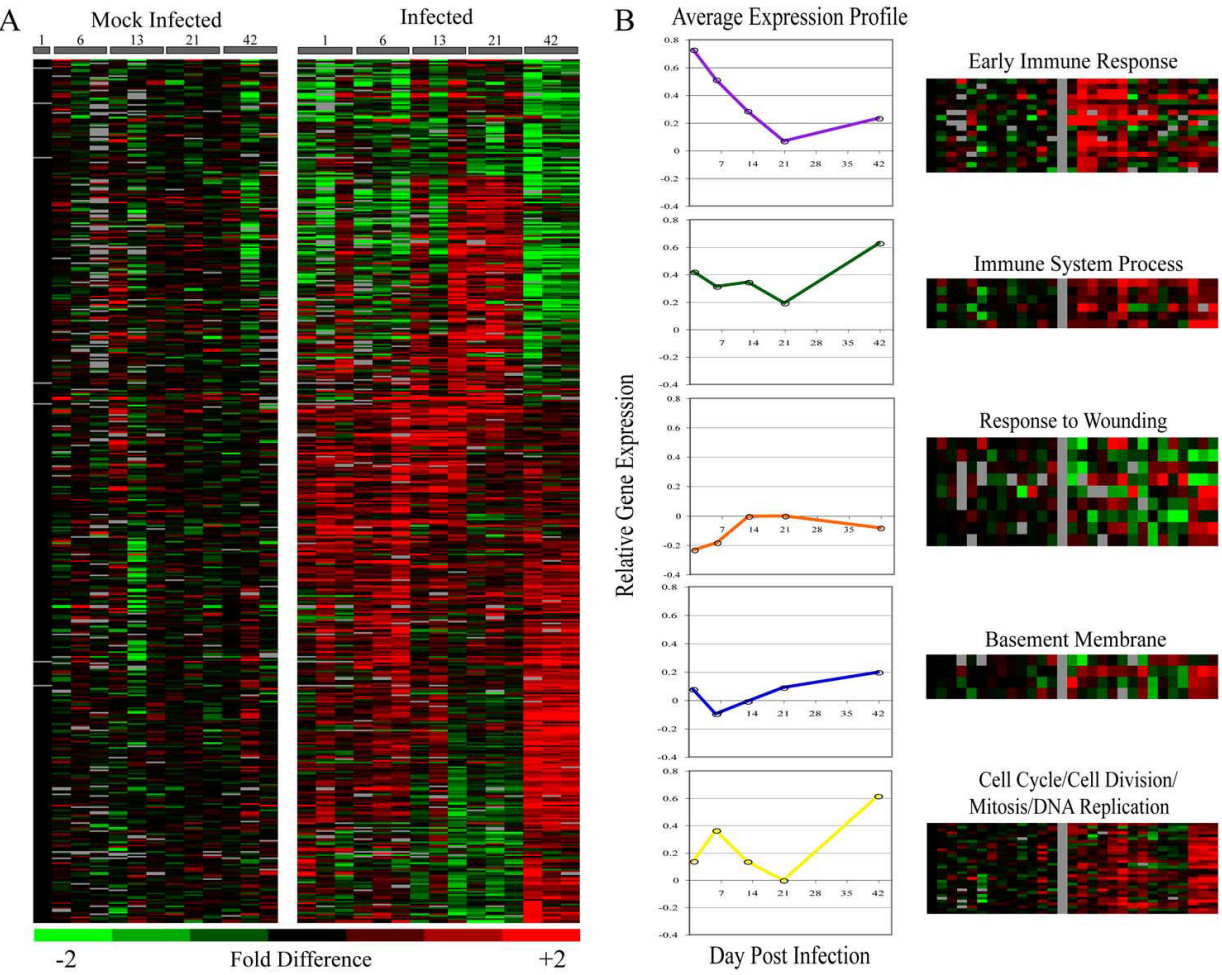
**NALT transcript analysis identifying genes that differentiate between mock-infected and pneumococcal-infected mice**. Comparisons were made between infected samples and mock-infected controls using Statistical Analysis for Microarrays (SAM) and the Fligner-Killeen Test for equality of variance. These analyses identified 112 transcripts that consistently differed during infection [SAM (FDR = 5%)] and the 358 transcripts (top 5%) that varied more during carriage as compared to control mice. **(A) **A union of the gene sets was clustered using a self-organizing map algorithm; each row represents a single gene, and each column a single animal. Black indicates the median level of expression, red indicates greater expression than the median, green less expression, and gray missing data. Numerically labeled horizontal gray bars above the samples indicate day post-colonization. **(B) **Expression profiles for the five functional categories identified. Heat maps illustrating the individual genes comprising each category are shown. An average expression profile for each category was calculated and plotted to illustrate the temporal characteristics of each category. Purple: Early Immune Response; Green: Immune System Process; Orange: Response to Wounding; Blue: Basement Membrane; Yellow: Cell Cycle/DNA Replication.

Complementing these ontological analyses, we visualized a union of both data sets, which is illustrated in Figure 2A. Samples were arranged by time post-infection and genes clustered using a Self Organizing Map (SOM) algorithm [23]. Visualizing the data in this way graphically illustrates the dynamic changes in gene expression during colonization as compared with mock-infection. These patterns roughly corresponded to gene subsets whose expression was induced early in the time course (1–13 days), during carriage (13–21 days), and at later stages when the organisms were disappearing from the nasal cavity (21–42 days). We examined the genes associated with these temporal categories to identify additional biological functions potentially associated with colonization that weren't revealed in the GeneTrail analyses. A set of 16 genes with a marked increase in expression on Day 1 was characteristic of early immune responses. 13 of the 16 genes are known to be stimulated by a Type I Interferon Response. A few of these genes were identified by both the SAM and variance analyses (*irg1*, *ifit3*, and *socs3*). These 16 Early Immune Response genes are listed in Table 3. We extracted the data for these 16 genes, as well as all of the genes in each of the GO or KEGG categories identified by the GeneTrail analyses, and calculated an average expression value at each time point. These data were plotted to give an average expression profile for each functional category over the course of infection. Several of the categories, like Mitosis, Cell Division, Cell Cycle, Microtubule-based Processes, and DNA Replication had similar expression profiles. Since these categories correspond to related processes, they were combined into a single group. Figure 2B illustrates both the average gene expression profiles for the major functional categories associated with carriage and the heat maps of the individual members of these categories. We were thus able to map the temporal expression pattern of the biological themes associated with asymptomatic colonization. Transcripts associated with the Early Immune Response cluster were most abundant between Days 1 and 6-post infection, after which their abundance declined, whereas transcript abundance for most of the genes involved in Cell Cycle and DNA Replication did not increase until Day 6 and continued throughout the time course, with maximal differences seen at Day 42. The genes comprising the Immune System Process category showed more variability in the timing of their induction but were, on average, most highly induced midway through the time course. Alterations in the expression profiles for the Basement Membrane and Response to Wound Healing categories were more modest, with initial changes seen during the first two weeks followed by a return to baseline expression levels.

**Table 3:** Terms associated with innate immune responses, particularly those involving type I interferons.

**Early Immune Response**
*casp4*

*cd274*

*defb3*

*gvin1*

*ifi203*

*ifit1*

*ifit2*

*ifit3*

*irg1*

*oas1g*

*oas1l*

*oas2*

*s100a8*

*socs3*

*stat1*

*stat2*

### Validating microarray data and demonstrating the induction of type I interferon genes during pneumococcal carriage

**Figure 3:**
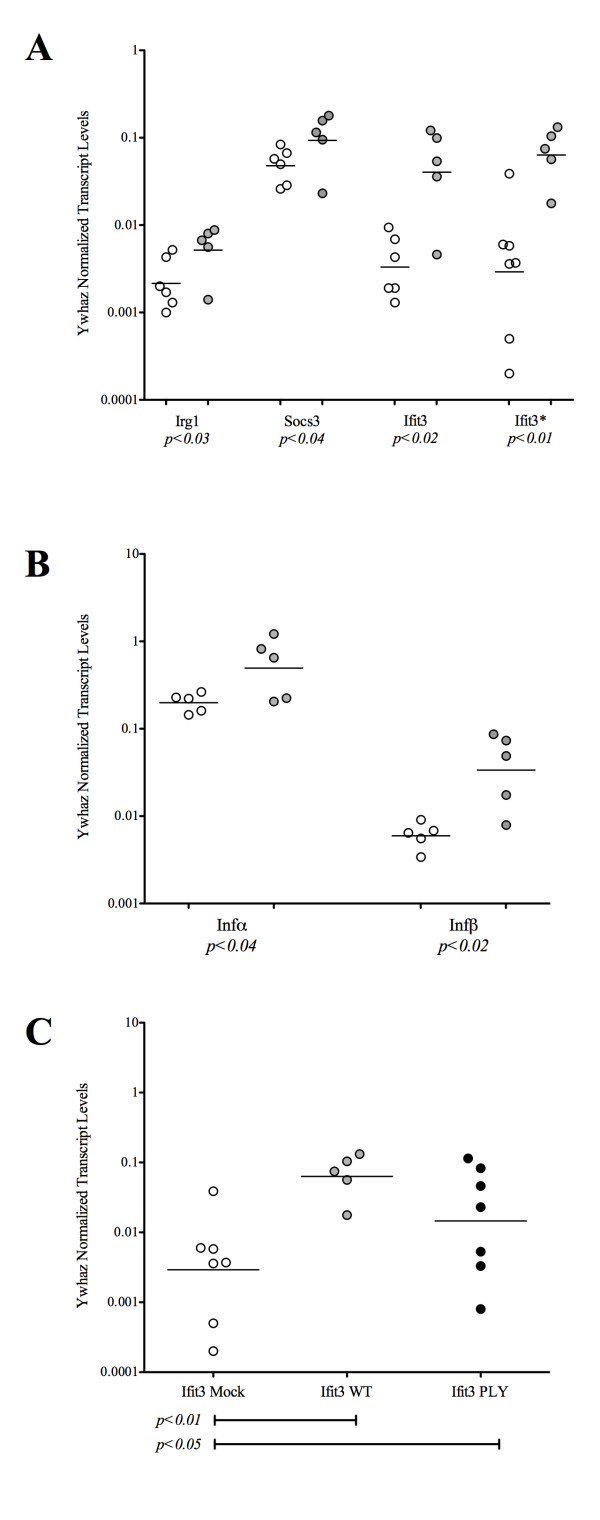
**Patterns of transcript abundance assessed by quantitative real time RT PCR as a function of pneumococcal colonization**. Validating the microarray data. Transcript abundance of (A) *irg1*, *socs3*, and *ifit3 *and (B) *infa *and *infB *was measured in mock infected and colonized mice using the original NALT RNA samples utilized for microarray analysis. These samples were collected from NALTs harvested within the first week of colonization. A second infection was established and RNA was isolated from NALTs of mock infected and pneumococcal colonized animals. Transcript abundance of *ifit3 *was measured in these samples (Ifit3*) (PANEL A). (C) A third infection time course was performed using mock infected, EJ1 (WT) colonized, and EJ5 (*ply*-) colonized mice. NALTs were isolated and the abundance of *ifit3 *transcript was assessed to determine to effect of pneumolysin on the Type I Interferon Response within the first week of colonization. Samples isolated on Days 1 and 3 post-colonization were not statistically different from each other using any of the primer sets. Therefore, they were combined into a single group. Open circles represent mock infected samples, closed gray circles represent EJ1 colonized samples, and black circles represent EJ5 colonized samples. Horizontal lines represent the geometric mean. *p *values were calculated using a 1-tailed Students T-test.

Of the several biological themes that emerged from the ontology and visual analyses of the data, we were most intrigued by the Early Immune Response category, as the genes comprising this category are associated with an inflammatory response. Given that our model is one of colonization and not disease, we chose to focus primarily on this category of genes. We validated the microarray findings by quantifying *irg1*, *ifit3*, and *socs3 *transcripts via quantitative reverse transcription PCR (qRT-PCR) using several of the original RNA samples as templates. In addition to these samples, we also isolated new RNA samples from the NALT of mice infected in an independent experiment (seven mock-infected and five infected) and assessed *ifit3 *transcript levels on Day 1 or Day 3 post-colonization. In all cases, we found that transcripts in the colonized mice were expressed at significantly higher levels than in the mock-infected control mice (Figure 3A).

Since a number of the genes identified by microarray analysis were annotated as being "interferon-induced", we wanted to determine which interferon(s) may participate in the regulation of these genes. We selected 10 of the RNA samples (five mock-infected and five infected) isolated at Day 1 or Day 6 from the original experiment to perform qRT-PCR for Type I (α and β) and Type 2 (γ) interferons. We were unable to detect message for Type 2 interferons in these samples (data not shown), however, we did observe that both α and β interferon transcript levels were significantly higher in colonized animals compared to the mock-infected control animals (Figure 3B) suggesting that Type I Interferons were involved in the induction of this set of genes.

### Ply, a TLR4 ligand, is not required for induction of type I interferon induced genes during pneumococcal carriage

The induction of Type I Interferon genes is accomplished by activation of a number of molecules that recognize pathogen associated molecular patterns (PAMPs), including members of the Toll-like receptor family (TLRs 3, 4, 7, 8, and 9), as well as intracellular receptors for viral RNA (RIG-I and MDA5). There is evidence that *S. pneumoniae *can activate TLR4 through pneumolysin (*ply*), a major virulence factor [24-27], and TLR9 [28,29]. TLR4 recognizes ligands at the cell surface, while TLR9 is located predominantly in endosomal compartments. Since we are interested in the interactions that occur between a bacterium and the mucosal surface during carriage, we wondered if TLR4 was involved in inducing a Type I Interferon response through its interactions with Ply. We infected mice with WT (EJ1) and an isogenic *ply*- (EJ5) strain of pneumococcus, isolated RNA at Days 1 and 3 from the NALT. As type I IFN-inducible genes have a more robust expression than type I IFN transcripts, we measured *ifit3 *transcript abundance as a downstream marker of Type I Interferon induction in the same NALT RNA. As expected, we observed a statistically significant difference in *ifit3 *transcript abundance between WT infected and mock infected animals (Figure 3C). Of interest, while the *ply*^- ^infected animals did show a statistically significant increase in *ifit3 *transcript as compared to the mock infected animals, it was not as great as that of WT infected animals, suggesting that Ply may contribute in part to induction of Type I Interferons and downstream signaling. The difference in *ifit3 *transcript between WT and *ply*- was not statistically different (*p *≤ 0.18).

## Discussion

### General

A thorough understanding of the complex relationships established between mucosal microorganisms and their hosts has been difficult to achieve. These interactions often occur without noticeable phenotype, which makes it challenging to study in the laboratory. In addition, the lack of any overt host response has led to the assumption that these interactions are benign from an immunological perspective, and have thus received little attention.

Here we present a comprehensive genome-wide view of the host response to asymptomatic pneumococcal carriage demonstrating that it is a dynamic process characterized by increased expression of a set of early inflammatory responses including induction of a Type I Interferon response, and the production of several antimicrobial factors. These initial responses are down-regulated within the first 2–3 weeks and are followed by increases in transcripts associated with immune system processes that affect T cell development and activation as well as those involved in cell cycle control. These data suggest that nasal carriage of *S. pneumoniae *results in an initial subclinical inflammation, which is insufficient to prevent colonization, followed by other activities related to T cell activation, wounding, basement membrane remodeling, and cell proliferation. These subsequent physiological processes presumably contribute to the resolution of pneumococcal carriage in the nasopharynx. Our results are consistent with recent studies suggesting that in contrast to "all or nothing" responses, the immune system is constantly and actively engaged in carefully calibrated responses to microbial/mucosal interactions. This finely tuned and complex spectrum of responses enables the host to strike a balance between controlled inflammation to eliminate potential pathogens and development of tolerance to the normal microbial flora.

### The early immune response in pneumococcal carriage

The up-regulation of many genes involved in early inflammatory responses upon pneumococcal colonization is striking considering the absence of clinically obvious symptoms. Of particular interest was the induction of Type I Interferons and interferon-induced genes. The critical role that Type I interferons play in establishing an antiviral state has been well documented over the last several decades, and there is accumulating evidence suggesting that these cytokines are also important in antibacterial defense against intracellular bacterial infections [30-33]. However, there has been little data regarding the potential role for Type I interferons in bacterial infections presumed to be extracellular [34-36]. In mice, the majority of Type I interferons are encoded by at least 14 interferon α-subfamily genes and a single interferon β gene. We determined that Type I interferons are expressed at higher levels in pneumococcal-colonized animals compared to the mock-colonized control animals by qRTPCR using a primer set specific for interferon beta and a set that amplifies several members of the interferon alpha family (Figure 3B). Once secreted from the cell, Type I IFNs bind to their cell surface receptor complex (INFAR) leading to the activation of the JAK/STAT signaling pathway. This signaling pathway in turn regulates the transcription of hundreds of Interferon Stimulated Genes (ISGs), whose products have anti-tumor, antiviral, immunomodulatory, and pro-apoptotic activities. This complex program of cellular responses permits affected cells to mobilize and orchestrate the innate immune system to swiftly achieve a state of resistance to intrusion by an infectious agent. Genes encoding components critical for controlling JAK/STAT signaling, including *stat1, stat2*, *socs3*, and *mapk1 *were identified in our microarray analyses as being induced in the colonized, but not in the mock-infected animals. Several ISGs were also found to be differentially regulated in the colonized animals including *oas1g*, *oas1l*, and *oas2, casp4*, *ifit1*, *2*, and *3*, *gvin1*, and *ifi203*. *oas1g*, *oas1l*, and *oas2 *belong to a family of pro-apoptotic, interferon-induced genes that are expressed in response to cell injury and viral infection. *casp4 *is an interferon-induced cysteine protease that has also been implicated in promoting cellular apoptosis [37,38]. *ifit1*, *ifit2 *[39], *ifit3 *[40], and *ifi203 *[41] are all IFN-a-inducible genes but have no described function. Several families of Interferon-induced GTPases have recently been described [42], and *gvin1 *is the prototype member of the newest family of very large IFN-inducible GTPases (VLIG) [43]. While the molecular basis for the antimicrobial/antiviral properties has not been elucidated for most of these GTPases, it is clear that each family shows a surprising level of pathogen-specificity in their activity [42].

In addition to the Interferon-induced genes, we also noted the upregulation of other genes known to be involved in early host defense in the infected mice. *s100A8 *encodes Calgranulin A, an intracellular calcium-binding protein and a member of a large family of pro-inflammatory molecules that are induced upon tissue damage. S100A8 is found in granulocytes, monocytes, and early differentiation stages of macrophages [44-46]. Interestingly, S100A8 is also induced in keratinocytes and epithelial cells under inflammatory conditions [47], and promotes neutrophil/monocyte recruitment to inflamed tissues, which has been documented during pneumococcal carriage [48].

Another host defense factor induced in our dataset is murine β-Defensin 3 (*defb3*). β-Defensins are a family of small cationic peptides produced predominantly by mucosal epithelial cells lining the respiratory, gastrointestinal and genitourinary tracts. When these cells sense microbial incursions, defensin peptides are released into the mucosal environment where they bind to and disrupt the cell membrane of microbes causing cell death. In addition to antmicrobial activity, many defensins also participate in cell signaling functions [49]. Bouskra *et al *report on some very interesting work involving a connection between murine β-Defensin 3 and the development of intestinal lymphoid tissue [50]. These authors demonstrate that genesis of isolated lymphoid follicles (ILFs) in the intestine is absolutely dependent upon the presence of the normal bacterial flora, and that the critical contribution of the microbiome in this process involves activation of CCR6 signaling by murine β-Defensin 3. Mice lacking either Defb3 or CCR6 are unable to form ILFs and as a consequence, they observe 100-fold increases in the total bacterial population in the intestine. These data suggest a major role for ILFs in maintaining intestinal homeostasis and demonstrate that normal intestinal flora and the immune system communicate through an innate detection system that includes β-Defensin 3 to generate adaptive lymphoid tissues. These observations are of great interest to us because, like the intestinal environment, the nasopharyngeal niche contains secondary lymphoid follicles within the NALT, which develop subsequent to microbial exposure [51]. The observation that *defb3 *is strongly induced at Day 1 in mice colonized with pneumococcus may be an early marker of lymphoid follicle formation in the NALT and may suggest an important role for β-Defensin 3 in promoting a specific adaptive immune response that ultimately helps to control the growth of *S. pneumoniae *in this niche.

Expression of *cd274 *(also known as *pd-l1 *or *b7-h1*) was also up-regulated in infected mice in the early time period. This gene encodes a ligand that, in combination with its receptor PD-1, delivers inhibitory signals that modulate the activation state of monocytes and CD4+ T cells [52] and is highly expressed on exhausted T cells during chronic viral infections [53]. An intriguing possibility to be further investigated is that PD-1 may be important for determining the balance between T cell activation, tolerance, and pathology during Streptococcal carriage.

### Induction of type I interferon response is not dependent upon the presence of pneumolysin, a TLR4 agonist

The induction of a Type I Interferon response in *S. pneumoniae *in the context of invasive disease has been described. Weigent *et al *[54] studied the role of interferons during interperitoneal (IP) pneumococcal infection. They showed that antibody depletion of Interferon α and β in mice resulted in marked decrease in survival after infection with the pneumococcus. As a corollary to this experiment, they also injected interferon α/β prior to infection and showed that this provided substantial protection against pneumococcal challenge. In mouse models of sepsis and meningitis, Mancuso *et al *[55] demonstrated that mice lacking the Type I Interferon receptor were more likely to suffer more severe bacteremia and die of infection that their WT counterparts. Our results show that these chemokines are involved not only in the response to invasive pneumococcal disease, but are also induced at the earliest stages in carriage. We were interested in determining the upstream signaling pathway(s) involved in induction of Type I interferons during pneumococcal carriage and focused on TLR4-mediated induction since it is known that pneumococcus activates TLR4 via the Ply hemolysin. Our results suggest that while Ply is not absolutely required for induction of a Type I interferon response, it may contribute to the magnitude of that response (Figure 3C) during carriage. There is other evidence to support such a role for Ply in Type I Interferon signaling. Rogers *et al *examined the effects of *S. pneumoniae *and an isogenic *ply*^- ^deletion strain on the gene expression of the THP-1 monocytic cell line [56]. THP-1 cells exposed to the WT but not the *ply*^- ^were able to induce expression of one of the Type I Interferon Receptor subunits (IFNAR2).

### Immune system process GO category

As the expression of the genes defining the early response to carriage decreased, we observed a concomitant and marked increase in transcript abundance of several genes, beginning on average at 3 weeks post-inoculation, that fell into the Immune System Process GO category (Figure 2B). Included in this category are *ptpn22*, *lilrb4*, *il1rl1*, *vav1*, *vav2*, and *flt3 *– all of which are involved in T cell development and/or activation and several of them contribute to reducing excessive inflammation [57-60]. T cells, in particular CD4^+ ^T cells, have recently been reported by several groups to play a critical role in resolving pneumococcal infection at both mucosal and systemic sites [8,9] and may be the major mechanism involved in naturally occurring, as opposed to vaccine-mediated, immunity to the pneumococcus [7].

### Response to wounding and basement membrane and DNA replication, cell division, and cell cycle GO categories

Genes within the Response to Wounding and Basement Membrane categories (Table 2) were modestly dysregulated in mice colonized with *S. pneumoniae*. Of particular interest was the identification of *ccl24 *(eotaxin-2), *entpd1*, and *darc*. Eotaxins (eotaxin-1 and eotaxin-2) have been identified as highly potent and selective chemoattractants for eosinophils in both mouse and humans [61-63]. While these proteins are redundant in function, it appears that eotaxin-1 plays a role in the early recruitment of eosinophils, while eotaxin-2 appears to be involved in eosinophil infiltration at later time points [64]. *entpd1*, which encodes an ecto-nucleoside triphosphate diphosphohydrolase 1 (also referred to as CD39) is a critical enzyme for hydrolysis of released ATP into adenosine. This activity has been shown to play a critical role in regulating neutrophil chemotaxis and cell migration [65]. *darc*, which encodes the Duffy antigen receptor for chemokines has been shown to bind inflammatory chemokines, however until recently, its in vivo function was unclear. Recently, Pruenster *et al *[66] demonstrated that DARC internalized chemokines mediating chemokine transcytosis, leading to apical retention of intact chemokines. This resulted in increased leukocyte migration across monolayers expressing DARC and enhanced chemokine-induced leukocyte extravasation in mice overexpressing DARC on blood vessel endothelia. Taken together, our data may indicate a role for chemotaxis of leukocytes and granulocytes, such as neutrophils and eosinophils, in the host responses to pneumococcal carriage.

The DNA Replication, Mitosis, Cell Division, Microtubule-based Process, and Cell Cycle GO categories pertain to processes involved in increasing cellular numbers (Tables 1 and 2). In almost every case, the genes that make up these categories are upregulated in infected mice beginning on Day 6 and show maximal expression at the end of the time course. These data imply that there is either an increase in proliferating cells in the NALT in response to the presence of the pneumococcus or an increase in cellular migration to the NALT. Definitive identification of cell types associated with the GO categories will require additional studies. However, our data do lend support to the idea that *de novo *proliferation of cells is occurring in the NALT, at least to some extent; at Day 6, we observed an increase in abundance of *mki67 *transcript. *mki67 *is expressed in actively cycling or recently divided cells but not resting cells [67-69]. In the absence of histological data on the cellular composition of NALT, we have attempted to address this important issue of *de novo *replication vs cell migration by comparing the expression profiles of our samples with published gene sets derived from distinct mouse tissues [70], which provide evidence correlating particular cellular compositions with expression data. The set of transcripts that increased in abundance in infected NALT samples between Days 6 and 13 (Figure 2) was enriched for genes associated with CD4+ T cells (*p *≤ 0.07). By Day 42, the association with lymphoid cells was both more significant and more generalized, with expression of genes associated with CD4+ T cells, CD8+ T cells, and thymus tissue significantly correlated with the patterns of gene expression we observed (*p *≤ 0.02, 0.02, and 0.01, respectively). Messages encoding immunoglobulin heavy chain subunits (Igh-1a and Igh-VJ558) were also more abundant at this time point and there was a highly significant overlap with a set of transcripts that were abundant in germinal center B cells following antigen-specific activation within the lymphoid tissue (*p *≤ 4E-6) [71].

Gene expression programs associated with activation or proliferation of specific cell types were also evident in our transcript data and Gene Ontologies. For example, transcripts more abundant at Day 13 in infected animals were associated with activation of murine macrophages in response to LPS and/or IFNγ but not the TH2 cytokine IL4 (*p *≤ 8E-6) [72], which may signify an important role for this cell type in the response to pneumococcal colonization. In addition, the identification of expression patterns associated with T cells and proliferation in our data is consistent with a recent report that examined histology and T cell proliferation in the NALT of mice infected with Group A Streptococcus (GAS) [73]. The authors report that GAS antigen-specific T cell priming, followed by a robust proliferation of T cells, occurred in the NALT within 4 days post-colonization. Re-challenge of these same mice with GAS drives the differentiation of effector T cells that express primarily T helper 1 (Th1) pro-inflammatory cytokines. Taken together, our observations and analysis prompt us to speculate that an early IFN-driven innate host response may lead to a TH1-type adaptive immune response. The presence of proliferation-associated transcripts as long as 6 weeks after infection in our dataset suggests that detailed histological and functional studies may reveal sustained alterations in the local immune environment that influence the response to subsequent infection with *Streptococcus *or other microbial agents.

It is important to recognize that our findings are based on examination of a single bacterial serotype colonizing a single mouse strain. We acknowledge that some of the host responses we observe in our study may be serotype-specific, but we believe that the vast majority of the responses will be elicited in a serotype-independent manner. Several lines of evidence indicate that factors other than capsule play a critical role in the development of the host response to pneumococci. For instance, in 1–2 year olds, the incidence of pneumococcal disease and nasopharyngeal colonization caused by different serotypes decreases sharply and simultaneously, suggesting that the host initiates a response that concurrently targets several different serotypes rather than mounting many individual immune responses that are serotype-specific. Secondly, experimental human carriage studies demonstrate that clearance of the colonizing strain is not temporally associated with the development of serotype-specific immunity [5,10]. Thirdly, several groups have now confirmed the critical involvement of cellular immunity (CD4+ T cells) in the clearance of pneumococcal carriage [7-9]. Since capsular polysaccharide is generally considered to be a T cell-independent antigen, there must be other serotype-independent bacterial factors that are important for host recognition of, response to, and clearance of the pneumococcus that are shared among many if not all colonizing *S. pneumoniae *isolates.

The enrichment of these 5 major functional categories in the infected animals provides a picture in which the nasopharyngeal tissue responds to the presences of pneumococci, and presumably other infection agents, with a closely regulated and specific arm of the inflammatory response that includes anti-microbial peptides. This process is undoubtedly an attempt to preserve and sustain an intact epithelial barrier.

For much of the past century, research on the pneumococcus has focused on understanding how this organism causes overt disease and elucidating the bacterial factors involved in this process. Our understanding of the host/microbe relationship has become more nuanced over time; we now appreciate that most of these interactions do not result in disease, and even those that do often do so only in certain hosts, highlighting that the outcome of a microbial/host interaction – that of disease or health – cannot be perfectly predicted simply by the initiation of that interaction. Rather, the consequences of these associations are determined by a complex and dynamic series of interactions. Furthermore, emphasis on disease neglects an important biological facet of the pneumococcal/host relationship: the human nasopharynx is the only known reservoir for this organism. Given that this bacterial/host interaction is essential for maintaining *S. pneumoniae *in human populations, refocusing some of our attention on the most frequent outcome of colonization, that of asymptomatic carriage of the mucosal surface, will likely lead to a better understanding of how this microbe interacts with its host.

Since carriage causes no obvious clinical symptoms, determining its actual impact on the biology of an animal has been difficult to ascertain. Nevertheless, there has been recent interest in dissecting the relationship established during carriage from both the bacterial as well as the host perspective. Several groups have conducted studies that examine the bacterial response to pneumococcal colonization *in vitro *and *in vivo *[9,74-81]. Studying the host response to carriage is more challenging because modeling the human host nasopharyngeal niche *in vitro *or *in vivo *is difficult. A few groups have conducted experiments examining the effects of *S. pneumoniae *cultures or culture extracts on explants of human nasopharyngeal and adenoid tissues in culture [82-84]. However, the fragile nature of the tissue samples necessitated very limited exposure time to *S*. *pneumoniae *and/or its products and focused predominantly on the bacterial impact on ciliary beat frequencies and epithelial cell integrity. Other studies have utilized mouse models to determine the contribution that particular host factors have on colonization and how they impact carriage by measuring serum antibody levels to pneumococcal antigens and assessing the ability of different mouse mutants to clear the pneumococcus from the nasopharynx [7,9,10,85]. These types of studies have contributed valuable insight regarding specific host proteins involved in pneumococcal carriage, but they reveal only a limited dimension of the host response to carriage. The development of genomic tools, such as microarray technology, has facilitated a global examination of this process. Microarry technology allows for the simultaneous examination of the transcriptional profile for every gene in a genome, and is particularly well suited to identify genes for which the impact of a specific process or condition, such as asymptomatic carriage, is unknown. Nelson *et al *[86] performed a microarray-based analysis on mice asymptomatically infected with *S. pneumoniae *and examined differences in gene expression in the epithelium overlying the turbinates between colonized and mock-colonized animals at a single time point, three days post-inoculation. In doing so, they identified one gene that differentiated pneumococcal-colonized animals from non-colonized animals.

The strength of our study lies in the examination of multiple time points over a six week period. This allowed us to investigate not only the earliest interactions between microbe and host, but also the changes that occur in the host while the organism is being carried subsequent to its clearance by the immune system, revealing the dynamic nature of the carrier state.

## Conclusion

This study provides a more subtle view of the host/microbe relationship as compared with the usual overwhelming systemic infection achieved in the laboratory by direct inoculation of the pneumococcus beyond the epithelial barrier. Our data suggest that the usual response to the presence of the pneumocccus in the nasopharynx is a controlled inflammatory response involving Type I Interferon signaling and appears to involve host physiological processes such as response to wounding, basement membrane remodeling, and increasing cellular numbers to maintain an intact epithelium and eventually a preventive adaptive immune response.

## Methods

### Bacteria and growth conditions

EJ1, a streptomycin-resistant derivative of D39 serotype 2, and EJ5, a streptomycin and spectinomycin-resistant EJ1 derivative deleted for pneumolysin (*ply*-) [87], were used in this study. Both strains were grown in brain heart infusion (BHI) broth (Difco), and on tryptic soy agar (TSA; Difco) supplemented with 5% defibrinated sheep blood (Hemostat Laboratories, Dixon, Calif.) at 37°C in 5–10% CO_2_. Both broth and agar media were supplemented with streptomycin (200 μg/ml) and/or spectinomycin (200 μg/ml) as needed. Agar medium also contained oxolinic acid (5 μg/ml) and colistin (10 μg/ml).

### Infection of mouse nasopharyngeal cavity, isolation of NALT, and plating viable counts

10–12 week old female BALB/c mice (Jackson Laboratories, Bar Harbor, Maine) were used during this study and were housed and cared for in accordance with Stanford University's Administrative Panel on Laboratory Animal Care (APLAC) protocols. To establish carriage, streptococcal strains were grown to an OD_600 _= 0.4, corresponding to a density of 1 × 10^8 ^CFU/ml. Cultures were centrifuged at 7,500 *g *for 10 min at 4°C. Cells were washed once in sterile phosphate buffered saline (PBS) and resuspended in PBS to a density of ~2 × 10^10 ^CFU/ml. Mice were lightly anaesthetized by exposing them for ~20 seconds to an Isofluorane-saturated cotton ball in a closed container. For the original time course experiment, mice were inoculated intranasally with either 10 μl of the above bacterial suspension (2 × 10^8 ^CFUs) (n = 15) or 10 μl PBS (n = 15). At 1, 6, 13, 21, and 42 days post inoculation, 3 mice from each group were sacrificed by CO_2 _asphyxiation and the nasal associated lymphoid tissue (NALT) was excised as described [12]. In brief, the skin and mandible were removed from each mouse head. Mice were pinned to a board face up and a dissecting scope was used for palate removal. The hard palate was excised using a #11 scalpel (Feather Disposable). Forcepts were used to carefully pull back the palate, exposing the underlying bilateral lobes of NALT. This tissue, which included the NALT, the overlying epithelial layer, and the hard palate was placed in a cryovial and flash frozen in liquid nitrogen. Samples were stored at -80°C. Subsequent to NALT excision, the central incisors were removed with a razor and a coronal section was cut through the skull at the tympanic bulla to remove the brain. The remainder of the head was homogenized in PBS and plated to determine bacteria load in the infected mice. Carriage was not accompanied by bacteremia as determined by plating blood samples obtained by cardiac puncture for viable counts. Similar methods were used for follow up experiments except that mice were sacrificed at Days 1 and 3 post-establishment of carriage.

### RNA preparation

Total RNA was isolated from the NALT samples using the MELT Kit (Catalog # AM1983) following the manufacturers recommendations (Applied Biosystems/Ambion, Austin, TX). Each RNA sample was subjected to spectroscopy and gel analysis. RNAs determined to be of high purity (260/280 ~1.8) and intact by gel analysis were used for microarray analysis (n = 28) or quantitative real time RT PCR.

### Microarray hybridization

Sample RNA transcripts and a standard reference RNA (Universal Mouse Reference RNA, Stratagene, La Jolla, CA) were amplified using the MessageAmp aRNA amplification kit (Ambion, Austin, TX) in the presence of amino allyl-labeled dUTP. Cy5 dye was subsequently conjugated to the experimental sample aRNAs, while Cy3 dye was conjugated to the reference aRNA. Equivalent amounts of sample and reference aRNAs were combined and hybridized to the Mouse Exonic Evidence Based Oligonucleotide (MEEBO) array [88]. Arrays were washed and then scanned using a GenePix 4000A scanner (Axon Instruments, Foster City, Calif.), and images were analyzed with GenePix Pro software. The raw data were loaded into the Stanford Microarray database [89]. The data are publicly available both in the Stanford Microarray Database and in the NIH Gene Expression Omnibus [90].

### Microarray data filtering and analysis

Data were filtered to include only spots that met the following criteria for at least 80% of the samples tested: signal intensity 2.5-fold above background in either the Cy5 (sample) or Cy3 (reference) channel, and a regression correlation for the two channels of at least 0.6 across each measured element. A normalization factor was applied so that the mean log_2 _ratio for each array (sample) was zero, and data for each spot were then median-centered across all observations. Transcripts that differed in abundance in control and infected mice were identified using Significance Analysis of Microarrays [15]; control and infected samples from each time point were treated as matched pairs. The Fligner-Killeen test for equality of variance [16] was used to identify transcripts with more variable expression in the infected mice than the control mice. Selected data from infected samples were normalized to the average of the day-matched control samples, organized using a Self Organizing Map (SOM) algorithm [23], and visualized using Java Treeview [91]. The data files for each array are publicly available at the Gene Expression Omnibus (GEO) database repository  (GSE 16803).

The GeneTrail software program [17,18] was used to identify Gene Ontology (GO) and Kyoto Encyclopedia of Genes and Genomes (KEGG) categories enriched in specified subsets of the data [20,92].

### Quantitative real time RT PCR

Differential gene expression identified by microarray analysis was validated by determining the relative quantities of several gene transcripts from infected and uninfected mice using real time RT PCR. Several recent studies have demonstrated that there is no one universal reference gene that has constant, stable expression in all different tissue types [93,94]. Thus, to identify an appropriate internal control for these experiments, we evaluated the expression of a set of commonly used control genes [94], and identified *ywhaz *(Accession #NM_011740) as a gene with low variance in expression across the entire sample set, and no difference in expression in the control and infected mice. PCR primers were designed and used for qRT PCR on a panel of representative test samples from both uninfected and infected mice. High Capacity cDNA kit (Applied Biosystems, Foster City, CA, USA) was used to generate first strand cDNA according to manufactures recommendations. Briefly, 2 μg of either Mouse Universal Reference or at least 0.5 μg of Sample total RNA were used as template for reverse Transcription. The reactions were run in an MJ Research Tetrad Thermal Cycler tracking the lid temperature (2°C above) as follows: 25°C/10 min; 37°C/120 min; 85°C/5 seconds. cDNA samples were purified using Zymo-5 DNA clean up and concentration kits (Zymo Research, Orange, CA). cDNA concentration and purity were assessed using a Nanodrop. PCR reactions were prepared using the Power SYBR Green PCR Master Mix (Applied Biosystems). Amplifications were performed on a AB 7300 real time machine under the following conditions: 95°C/10 min; 95°C/15 seconds; 55°C/60 seconds; return to step 2 for 44 more times. Primers used in this study are listed in Table 4.

**Table 4:** Primers used in this study

**Primer Name**	**Sequence 5' → 3'**
Ywhaz_F	CACAGCCTCCCCTCATCCT

Ywhaz_R	GGGAGACGGTGACAGACCAT

Socs3_F	GCCACCTGGACTCCTATGAGAA

Socs3_R	TCTGACCCTTTTGCTCCTTAAAGT

Ifit3_F	GGGAGAATGTGCTGAAAAAAGC

Ifit3_R	GGAGTCAGGGAGAGAAAGCAGTT

Irg1_F	TTTTTCTTTCCACACAGAGCCTTA

Irg1_R	ACTGCTTCACCACCCCAAGT

InfA2_F	GTGAGGAAATACTTCCACAG

InfA2_R	GGCTCTCCAGACTTCTGCTC

InfB2_F	CAGCTCCAAGAAAGGACGAAC

InfB2_R	GGCAGTGTAACTCTTCTGCAT

InfG_F	GAACTGGCAAAAGGATGGTGA

InfG_R	TGTGGGTTGTTGACCTCAAAC

Universal Mouse Reference RNA (Stratagene) spiked with RNA known to contain Type I interferon messages was used to establish a standard curve, which was highly reproducible between assays (Pearson correlation coefficient >0.998 over a four-log (base 10) range). Relative abundance of the target transcripts was calculated by comparison to the standard curve, and normalized to *ywhaz *expression levels.

### Confocal immunofluorescent microscopy

For immunofluorescence microscopy, samples were fixed with 2% paraformaldehyde in 100 mM phosphate buffer (pH 7.4) for 1 h, and were permeabilized in PBS 1% saponin 3% bovine serum albumin. After incubation with appropriate antibodies/probes, we mounted intact tissue in Vectashield mounting medium (Vector Laboratories, Burlingame, California, United States and imaged the stained tissues without prior embedding and sectioning. Samples were imaged with a confocal microscope (Bio-Rad) by taking optical sections at 0.5-μm resolution. Figures were assembled with Photoshop software (Adobe, San Jose, California, United States).

*S. pneumoniae *were detected by incubation of samples with 1:500 dilution of polyclonal IgY antibodies (chicken) (Aves Labs, Tigard, Oregon). Host cell DNA was visualized using toto-3 (Molecular Probes). NALT tissue contains mucus-producing goblet cells. To visualize NALT, we used 1:300 dilution of the Ulex europeas Agglutinin 1 (UEA 1) lectin, which preferentially binds to goblet cells. Anti-IgG 1:500 dilution of Alexa-fluor conjugated chicken antibodies were used for secondary detection of the anti-pneumococcal IgY (Molecular Probes).

## Authors' contributions

EAJ and SF conceived of the study. EAJ designed and executed all experiments, analyzed data, and drafted the manuscript. SJP participated in the statistical analyses of the microarray data and contributed to drafting the manuscript. SF provided valuable input and support at all stages of the project. All authors read and approved the final manuscript.

## Supplementary Material

Additional file 1 **Gene List resulting from SAM analysis**. List of gene symbols and description as well as the microarray oligo ID for genes whose expression consistently differed between infected and mock-infected mice over the entire time course. Click here for file

Additional file 2 **Gene List resulting from Variation analysis**. List of gene symbols and description as well as the microarray oligo ID for genes whose expression varied most between infected and mock-infected mice over the entire time course. Click here for file
